# Epithelial Cell Adhesion Molecule Is an Accurate Target for Fluorescence Guided Imaging of Lymph Nodes

**DOI:** 10.1007/s11307-025-02058-5

**Published:** 2025-10-14

**Authors:** Kelly Anne McGovern, Katherine O. Welch, Ryan Krouse, Michael Brown, Lydia Chen, Kevin Guo, Jeffrey Huang, Jake Mlakar, Edward J. Delikatny, Viktor Gruev, Paul Zhang, Sunil Singhal

**Affiliations:** 1 Division of Thoracic Surgery, Hospital of the University of Pennsylvania, Philadelphia, PA 19104 USA; 2 Department of Radiology, Hospital of the University of Pennsylvania, Philadelphia, PA 19104 USA; 3 Department of Engineering and Computer Engineering, University of Illinois at Urbana-Champaign, 306 N Wright St, Urbana, IL 61801 USA; 4 Department of Pathology and Laboratory Medicine, Hospital of the University of Pennsylvania, Philadelphia, PA 19104 USA

**Keywords:** Fluorescence-guided surgery, Lymph nodes, Lung cancer, Intraoperative molecular imaging

## Abstract

**Purpose:**

Lymph node (LN) excision is critical in oncologic surgery to provide important therapeutic and diagnostic information. LN evaluation helps in staging cancers, predicting prognosis and improving survival. The ultimate wish of a surgical oncologist would be to localize and dissect all pathologically positive LNs while avoiding the morbidity of removing true negative LNs. The goal of our study was to identify a reliable marker for intraoperative molecular imaging of LNs with cancer cells from non-small cell lung cancer versus a LN without.

Procedures.

We identified Epithelial Cell Adhesion Molecule (EpCAM), a membrane protein normally expressed in epithelial tissues including lung. We performed immunofluorescence staining on human specimens with a conjugated anti-EpCAM monoclonal antibody.

**Results:**

Fluorescence was significantly higher in LNs with metastases as shown in 48 positive LNs from patients with resected primary lung cancer. There was high fluorescence in both hilar and mediastinal LNs, and in all primary tumor histologies.

**Conclusions:**

EpCAM may be useful for the surgical oncologist for intraoperative molecular imaging of positive LNs from lung cancer.

**Supplementary Information:**

The online version contains supplementary material available at 10.1007/s11307-025-02058-5.

## Introduction

Lymph node (LN) excision is critical in oncologic surgery to provide important therapeutic and diagnostic information. LN evaluation helps in staging cancers, predicting prognosis and improving survival [1–3]. The procedure for harvesting LNs during a cancer surgery varies for different cancer types such as lung, breast, gastric, colon, and thyroid.

For example, in lung cancer surgery, some surgeons perform a lobe-specific approach to LN dissection, resecting only LNs contiguous to the resected lung lobe; however, using this method, 6% of patients can have missed LNs that harbor cancer cells (“positive” LNs) because they exist in non-contiguous regions [4]. Missing positive LNs results in inaccurate staging, failure to offer adjuvant treatment, and decreased survival rates. The most recent guidelines, according to the European Society of Thoracic Surgeons (ESTS), advise performing a systematic LN dissection, including at least 3 mediastinal LN stations, requiring the excision of stations 7 (subcarinal area), 10 (hilar LNs) and 11 (interlobular LNs) [5, 6]. These differences in practice highlight a major challenge for surgical oncologists to intraoperatively identify LNs that harbor cancer cells.

The ultimate wish of a surgical oncologist would be to localize and dissect all positive LNs while avoiding the morbidity of removing true negative LNs. However, this is not possible because positive and negative LNs have similar color, shape, hardness and consistency. The current protocol is a non-specific aggressive dissection of all LN stations to reduce false negatives, but this increases the morbidity to the patient because of potential damage to lymphatic channels, nerves, and blood vessels.

Intraoperative molecular imaging (IMI) has emerged as a new technology that enhances cancer visualization during surgery by utilizing targeted fluorescent contrast agents that selectively accumulate in tumors. Once the fluorescent contrast agent has collected in the tumor, a wavelength specific excitation laser and camera system can be used to enhance visualization of the lesion [7, 8]. It has been adopted for gliomas, ovarian cancer, breast cancer, and lung cancer, for the purposes of improving tumor localization, assessing margins, and detecting synchronous disease [8–12] Despite these advancements, no currently available contrast agent effectively distinguishes lymph nodes with cancer cells from normal LNs.

The goal of our study was to identify a reliable marker for intraoperative imaging to distinguish a negative versus positive LN. After a broad screen of many biomarkers to distinguish negative versus positive LNs, we identified Epithelial Cell Adhesion Molecule (EpCAM, CD326). EpCAM is a type I transmembrane glycoprotein that contributes to cell adhesion, signaling, migration, proliferation, maintenance of organ morphology [13–15]. It is normally expressed in epithelial tissues including in lung, stomach, pancreas, kidney, small intestine, and elsewhere throughout the body. It has been proposed as a tumor-associated antigen due to its high level of expression in rapidly growing epithelial tumors. In tumors, its functions include activation of cell proliferation, promoting oncogenesis via cell–cell signaling, resistance to apoptosis, regulation of epithelial-mesenchymal transition and metastasis, and formation of exosomes [16].

Although EpCAM is a well-accepted marker for lung cancer, to our knowledge it has not been characterized in the context of intraoperative imaging for normal LNs in the thorax and pulmonary metastases to LNs. We hypothesized that EpCAM would be a reasonable imaging target to accurately identify LNs with metastatic lung cancer cells and differentiate them from LNs without metastatic lung cancer cells. In this study we determine the imaging characteristics when targeting EpCAM in positive LNs compared to negative LNs in patients with lung cancer.

## Methods

### Assessing EpCAM Expression in Human Lung Cancer Metastases by Immunohistochemistry

Tissue specimens were obtained from 48 LNs from 18 patients with LN metastases from primary lung cancers who had undergone surgeries at the Hospital of the University of Pennsylvania. Patients were excluded if they had primary tumor histology other than lung cancer or did not have positive LNs found on postoperative pathologic assessment. All patients were operated on at the Department of Thoracic Surgery, Hospital of the University of Pennsylvania, and had given written informed consent. The study protocol was approved by the University of Pennsylvania Institutional Review Board and was conducted in accordance with the Declaration of Helsinki.

Samples were prepared and immunostained for EpCAM using anti-EpCAM monoclonal antibody (mAb) (Cell Signaling Technology, Danvers, MA; catalog number 2929) applied to formalin-fixed, paraffin-embedded 5-micron thick sections. The sections underwent a process of deparaffinization, rehydration, and washings in xylene, graded alcohols, and distilled water. The sections were placed in 10 mM citrate buffer at pH 6 with subsequent microwave antigen retrieval procedure. The slides were incubated with the mAb at a 1:100 dilution. The antigen–antibody reaction was visualized using the avidin–biotin-peroxidase complex with diaminobenzidine as the chromogen. The slides were counterstained with hematoxylin.

### Assessing EpCAM Expression in Human Lung Cancer Metastases by Immunofluorescence

The unstained slides from patient positive and negative LN samples were evaluated for fluorescence with an anti-EpCAM mAb conjugated to AlexaFluor750 (excitation 750 nm, emission 776 nm) (Novus Biologicals, Minneapolis, MN; catalog number NBP2-54348AF750). The sections underwent a process of deparaffinization, rehydration, and washings in xylene, graded alcohols, and distilled water. The sections were treated as described above and incubated with the mAb a 1:100 dilution. Slides were mounted with ProLong Gold Antifade Reagent containing the nuclear dye 4',6-diamidino-2-phenylindole (DAPI) (Fisher Scientific, Waltham, MA). Fluorescence was imaged with a Leica DM6 B fluorescence microscope (Leica Microsystems, Wetzlar, Germany) with a Cy7 filter, as recommended for AlexaFluor750 conjugates.

### Post Hoc Image Analysis and Statistics

Post hoc image analysis was conducted with ImageJ [17]. Using H&E samples, the proportion of the area containing tumor cells compared to the overall area of the LN was calculated to obtain percent of cancer cells out of all cells in the LN, or percent tumor burden. On immunofluorescence samples, after correcting for distant background fluorescence, the mean fluorescence intensity (MFI) of the tumor and the background (normal tissue) were measured to calculate a signal-to-background ratio (SBR) for each sample. A SBR greater than 2 was the designated cutoff for sufficient fluorescent contrast to delineate tumor from background within each sample. The positive LN signal was compared to negative LN signal by comparing MFI of the two samples, termed tumor-to-normal ratio (TNR). To maintain consistency, all fluorescence values below 1 were adjusted to a minimum value of 1.

### Chart Review and Subgroup Analyses

Under an approved University of Pennsylvania IRB-approved protocol, chart review was performed using electronic medical record. For patients with LNs with pulmonary metastasis from lung cancer, information was collected including relevant patient and tumor characteristics. Subsets of the positive and negative LN populations were analyzed to determine if EpCAM positivity correlated with clinical characteristics. Anatomic location of positive LNs was analyzed to determine correlation with EpCAM expression. LNs were stratified by hilar location and mediastinal location. Primary tumor histology was also analyzed to determine correlation with EpCAM expression of positive LNs. Event-free survival was captured, with events including recurrence, relapse, or death.

### Statistical Analysis

Statistical analysis was performed using GraphPad Prism 8 (GraphPad Software, CA, USA). Data analysis is stratified by LNs harboring pulmonary metastases compared to LNs without metastases. Patient and tumor characteristics are summarized using descriptive statistics or proportions. Between group analysis for tumor characteristics was performed using paired t-tests. A log-rank test was performed for event-free survival. P values less than 0.05 were considered statistically significant.

## Results

### Anti-EpCAM-AlexaFluor750 Distinguishes Metastatic and Benign LNs

Based on encouraging preclinical data, we aimed to confirm our findings in human samples with resectable non-small cell lung cancer. We identified a set of 18 patients who underwent pulmonary resection for lung cancer. Supplementary Table 1 describes characteristics of the included patients. Patients had a mean age of 57.9 ± 11.4 years and all patients had N1 or N2 stage disease on postoperative pathologic assessment. In the 18 patients, we obtained a total of 48 lymph nodes (LNs) that harbored cancer cells and 210 normal LNs without cancer cells. Each sample underwent anti-epithelial cell adhesion molecule (EpCAM) monoclonal antibody (mAb) immunofluorescence (IF) staining and was reviewed by a board-certified lung pathologist.

**Fig. 1:**
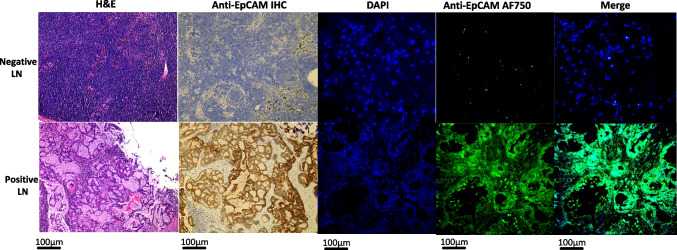
Representative immunohistochemistry and immunofluorescence images in negative lymph nodes (upper row) and positive lymph nodes (bottom row), showing increased fluorescence consistent with the presence of tumor cells in immunohistochemistry and Hematoxylin and eosin (H&E) staining. (EpCAM Epithelial Cell Adhesion Molecule, LN Lymph Node, DAPI 4',6-diamidino-2-phenylindole, IHC immunohistochemistry, AF750 AlexaFluor750)

Of the 48 LNs that harbored cancer cells, 100% of them had fluorescence to some degree which was indicative of EpCAM expression. As shown in Fig. 1, the areas of EpCAM expression in the positive LNs was consistent with areas of cancer cells in the LN.

### Quantification of EpCAM Fluorescence in Pulmonary Metastases

**Fig. 2:**
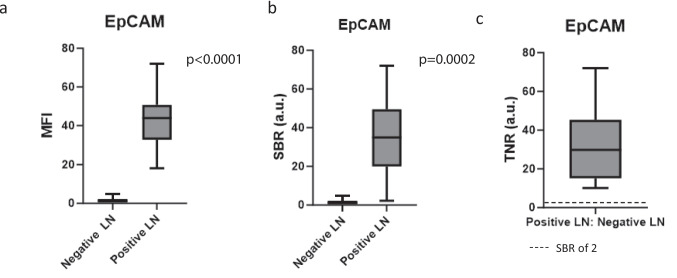
Quantification of EpCAM-targeted fluorescence. **a** MFI in positive LNs was 42.5 (IQR 32.7–50.8), significantly higher than in the negative LN specimens, which had MFI of 1.7 (IQR 1.0–2.1) (*p* < 0.0001). **b** The SBR was significantly higher in the positive LNs compared to the negative LNs (43.7 vs. 1.5, *p* = 0.0002). **c** When the MFI of positive LNs were compared to negative LNs from the same patient, the resulting TNR was 45.30 (IQR 31.1–61.4).

Next, our goal was to quantify the EpCAM fluorescence in positive versus negative LNs. Of the 48 positive LN specimens, the Mean Fluorescence Intensity (MFI), was 42.5 (IQR 32.7–50.8), significantly higher than the 210 negative LN specimens, which had MFI of 1.7 (IQR 1.0–2.1) (*p* < 0.0001) (Fig. 2a). The fluorescence was confirmed to correlate to areas of cancer cells within the LNs.

The Signal to Background Ratio (SBR) was significantly higher in the positive LNs compared to the negative LNs (43.65 vs. 1.48, *p* = 0.0002) (Fig. 2b). When the MFI of the positive LNs were compared to the negative LNs from the same patient, the tumor-to-normal ratio (TNR) was 45.3 (IQR 31.1–61.4). This was much greater than 2, the convention for clinically useful SBR, revealing a significant intra-patient difference between positive and negative LNs (Fig. 2c).

### EpCAM Fluorescence is Observed in Hilar and Mediastinal Lymph Node Metastases

**Fig. 3:**
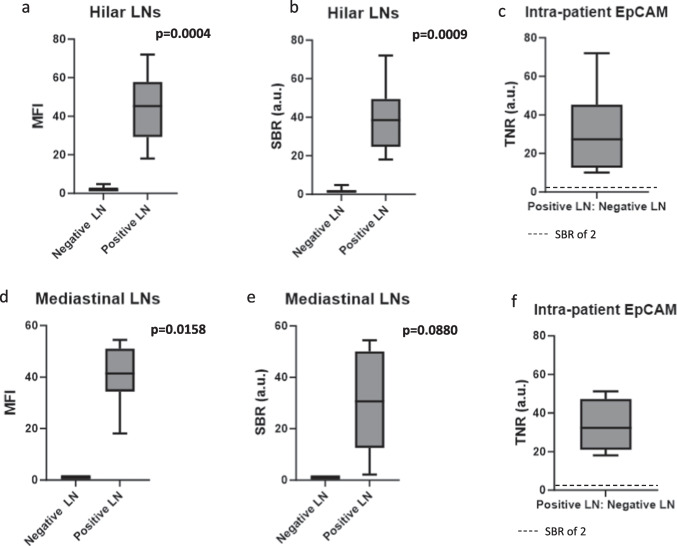
Quantification of EpCAM targeted fluorescence in proximal (hilar) vs distant (mediastinal) LNs. **a** In hilar LNs, the MFI was significantly higher in positive LNs compared to negative LNs (44.1 vs. 2.1, *p* = 0.0004). **b** The SBR was significantly higher in the positive hilar LNs compared to the negative hilar LNs (39.3 vs. 1.9, *p* = 0.0009). **c** When the MFI of the positive hilar LNs were compared to the negative LNs from the same patient, the resulting TNR was 31.5 (IQR 12.6–45.4), revealing a clinically significant intra-patient difference. **d** In mediastinal LNs, MFI was significantly higher in positive LNs compared to negative LNs (40.8 vs. 1.0, *p* = 0.0158). **e** Similarly, in mediastinal LNs, SBR was higher in positive LNs compared to negative LNs (28.9 vs. 1.0, *p* = 0.0880). **f** The intra-patient MFI ratio (TNR) was 33.5 (IQR 21.0–47.2).

We next sought to determine if there was any difference in EpCAM fluorescence in positive LNs that may be spatially close to or distant from the primary pulmonary malignancy. We used hilar LNs as a surrogate for a “close” LN because they were anatomically within the same lobe as the cancer. Then, we used mediastinal LNs as a surrogate for “distant” LNs because they were no longer invested with the pleural lining of the lung organ. Of 23 hilar LNs, the fluorescence as measured by MFI was significantly higher in the positive LNs compared to the negative LNs (44.1 vs. 2.1, *p* = 0.0004) (Fig. 3a). Similarly, the SBR was significantly higher in the positive LNs compared to the negative LNs (39.3 vs. 1.9, *p* = 0.0009) (Fig. 3b). When the MFI of the positive hilar LNs were compared to the negative LNs from the same patient, the TNR was 31.5 (IQR 12.6–45.4), again revealing a significant intra-patient difference (Fig. 3c).

Of 25 mediastinal LNs, the MFI (40.8 vs. 1.0, *p* = 0.0158) (Fig. 3d) and SBR (28.9 vs. 1.0, *p* = 0.0880) (Fig. 3e) were higher in the positive LNs compared to the negative LNs. When the MFI of the positive mediastinal LNs were compared to the negative LNs from the same patient, the resulting TNR was 33.5 (IQR 21.0–47.2) (Fig. 3f).

### EpCAM is Fluorescence is Observed in Lymph Node Metastases from Different Histologic Types

**Fig. 4:**
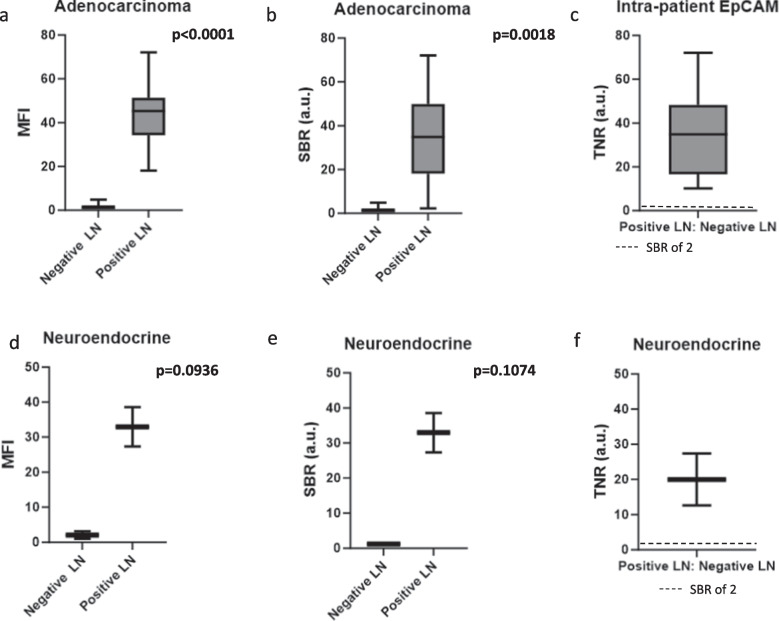
Quantification of EpCAM-targeted fluorescence in positive LNs with different primary tumor histologies. **a** MFIs of positive LNs from adenocarcinoma primary tumors were significantly different compared to negative LNs (43.8 vs. 1.6, *p* < 0.0001). **b** SBR was significantly different in positive LNs from adenocarcinoma primary tumors compared to negative LNs (34.6 vs. 1.6, *p* = 0.0018). **c** The intra-patient MFI ratio was 34.9 (IQR 16.6–48.3), revealing a significant difference between positive and negative LNs. **d** Of LNs from primary neuroendocrine tumors, the MFI (32.9 vs. 2.0, *p* = 0.0936) (**e**) and SBR (32.9 vs 1.2, *p* = 0.1047) were higher in positive LNs. **f** The intra-patient MFI ratio (TNR) was 20.0 (IQR 12.6–27.3), revealing a large intra-patient difference.

We then hypothesized that EpCAM expression may only correlate with histological subtypes of lung cancer. Thus, primary tumor histology was analyzed to determine correlation with EpCAM-targeted fluorescence of positive LNs including primary adenocarcinoma tumors (*n* = 42) and primary neuroendocrine tumors (*n* = 6). Of LNs from primary adenocarcinoma tumors, the MFI (43.8 vs. 1.6, *p* < 0.0001) (Fig. 4a) and SBR (34.6 vs. 1.6, *p* = 0.0018) (Fig. 4b) were significantly higher in the positive LNs compared to the negative LNs. When the MFI of the positive hilar LNs were compared to the negative LNs from the same patient, the resulting TNR was 34.93 (IQR 16.6–48.3) (Fig. 4c).

Of LNs from primary neuroendocrine tumors, the MFI (32.9 vs. 2.0, *p* = 0.0936) (Fig. 4d) and SBR (32.9 vs 1.2, *p* = 0.1047) (Fig. 4e) were higher in the positive LNs compared to the negative LNs, although statistical significance was likely limited by low sample numbers in this analysis. When the MFI of the positive hilar LNs were compared to the MFI of the negative LNs from the same patient, the resulting TNR was 20.0 (IQR 12.6–27.3), revealing a large intra-patient difference (Fig. 4f).

### EpCAM Fluorescence is Measurable Even at Low Tumor Burden in Lymph Nodes

**Fig. 5:**
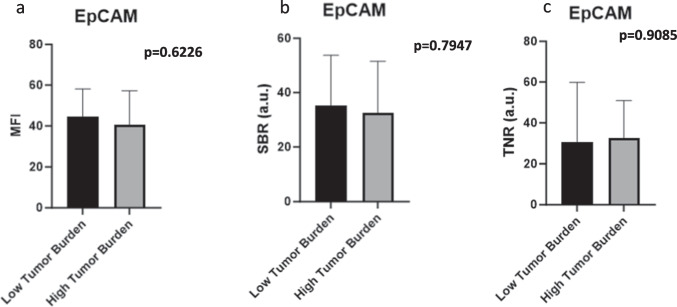
EpCAM-targeted fluorescence in relation to LN tumor burden. **a** There was no difference in MFI between positive LNs with micrometastases compared to those with macrometastases. **b** There was no difference in MFI between positive LNs with micrometastases compared to those with macrometastases.** c** There was no difference in TNR between positive LNs with micrometastases compared to those with macrometastases, revealing high sensitivity for low tumor burden.

Since lung cancer recurrence may often be secondary to micrometastases in LNs, our next goal was to assess whether fluorescence would be present in LNs with low tumor burden. We used tumor burden in a LN of less than 5% as a surrogate for micrometastases or “low tumor burden”, and tumor burden of greater than or equal to 5% as a surrogate for macrometastases or “high tumor burden”. When MFI was plotted against tumor burden, there was no difference in fluorescence between low tumor burden or high tumor burden LNs (*p* = 0.6226) (Fig. 5a). Similarly, there was no difference between SBR of low tumor burden and high tumor burden LNs (*p* = 0.7947) (Fig. 5b). When the MFIs of the positive LNs were compared to the MFI of the negative LNs from the same patient, acting as an intra-patient normalization, the resultant TNRs were similar between the low tumor burden and high tumor burden LNs (*p* = 0.9085), indicating that the fluorescence, even in micrometastases, is clinically significant (Fig. 5c).

### Event-Free Survival

**Fig. 6:**
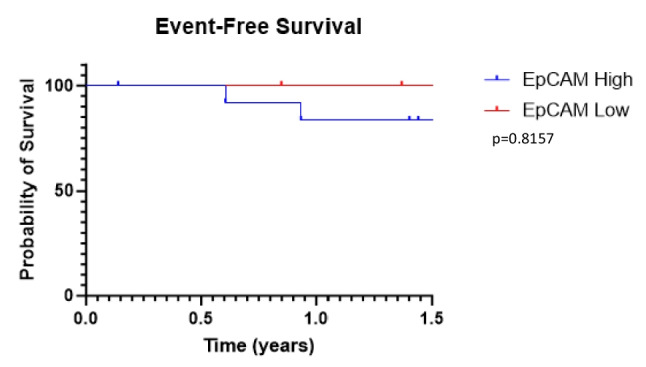
Event-free survival evaluated by MFI, as grouped by high EpCAM (upper three quartiles) and low EpCAM (lower quartile) revealed no significant difference

Event-free survival (EFS) was evaluated to see if EpCAM expression correlated to clinical outcomes. Patients were grouped by high EpCAM (upper three quartiles of MFI) and low EpCAM (lower quartile of MFI), and log-rank test was performed for EFS. There was no statistically significant difference in EFS between the EpCAM-high and EpCAM-low groups, although there was clear separation between the EFS of the two groups by 18 months after surgery (Fig. 6). This does suggest that higher tumor burden in lymph nodes correlates to poorer prognosis. Notably our study was not powered to evaluate survival, but given these exploratory results, with adequate powering for survival, high fluorescence from targeting EpCAM would likely correspond to worse clinical outcomes.

## Discussion

In this study, we report that targeting epithelial cell adhesion molecule (EpCAM) with a fluorescent probe in intraoperative molecular imaging (IMI) offers highly sensitive and specific imaging capabilities of lymph nodes (LNs) with minimal background fluorescence.

EpCAM-targeted fluorescence was significantly higher in both hilar LNs and mediastinal LNs compared to background and normal LNs. Since EpCAM is upregulated in cancer cells preceding the epithelial-mesenchymal transition and metastasis, it is not unexpected that both hilar and mediastinal LNs would have cancer cells and therefore high EpCAM fluorescence. Additionally, we observed significant EpCAM-targeted fluorescence in positive LNs from different primary tumor histologies, including adenocarcinoma and neuroendocrine tumors. As the tumor cells dedifferentiate and increase EpCAM expression as they become more advanced, it is not unexpected that EpCAM fluorescence would be increased regardless of primary tumor histology. Notably, the subgroup for neuroendocrine histology was small (*n* = 6), and warrants further investigation with a larger sample size to confirm this conclusion.

Thoracic surgeons continue to face the challenges of occult N2 disease and skip metastases, where the cancer cells spread to a LN farther from the tumor, bypassing the closer LNs. Recent conflicting data on the prognosis of skip N2 disease highlights the need for more systematic and complete LN dissections to ensure patients receive full adjuvant therapy when needed [5, 6, 18, 19]. Currently, cancer surgeons rely on visual and tactile cues to identify irregular nodules, and frozen section is used to confirm pathologic diagnosis; however, this may be time consuming and variable in its accuracy. These methods are helpful for primary tumors, though do not always provide the same utility for LN dissection. Frozen section of LNs is not always representative of the cancer spread to that area due to skip metastases [20, 21], and visual and tactile cues will not detect microscopic nodal metastases.

IMI, also known as fluorescence-guided surgery, utilizes injected fluorescent contrast-agents that target cancer cells [7, 22, 23]. IMI has emerged as a promising approach for intraoperative localization and margin assessment.^24^ IMI has shown success in enhancing resection of many solid tumors including ovarian, lung, glioma, and breast cancers [10, 11, 23–27]. Currently used molecular targets include cathepsins, annexins, and folate receptor [9, 26, 28–31]. Although these have been useful in accurately detecting primary tumors, there is not yet a target for LN metastases.

Cathepsins are lysosomal cysteine proteases involved in protein degradation, and they are found to be overexpressed by tumor cells and TAMs. VGT-309 which is a quenched activity-based probe that is activated by cathepsins, fluorescing where there is high cathepsin activity, such as the tumor microenvironment [26, 28]. Other non-cancerous inflammatory processes might also cause an increased cathepsin expression and could potentially be a cause of a false positive. Therefore, inflammatory or reactive LNs may have false positives without having cancer cells with this imaging agent.

Annexins, particularly annexin V, are used to target phosphatidylserine, a specific marker of apoptosis (programmed cell death). Annexin-based imaging agents useful for visualizing apoptotic or dying cells, including within tumors.^32^ Imaging with annexin-targeted probes is not tumor-specific; they bind to any apoptotic cell, not just cancer, which can lead to off-target signal, such as in inflamed or healing tissues. Annexin-based imaging probes would not be effective for imaging LNs which may have inflammation, resulting in a false positive.

Pafolacianine targets is the folate receptor alpha (FRα), a cell surface glycoprotein that is overexpressed in many cancers including non-small cell lung cancer (NSCLC). FRα expression is low in normal lung tissues, making it an excellent molecular target for IMI of NSCLC [9, 12, 31]. Although Pafolacianine is targeted to FRα, it may also bind to folate receptor beta (FRβ), another isoform of the folate receptor. This hinders IMI of LNs with cancer cells because macrophages express FRβ, and this expression is particularly prominent in tumor-associated macrophages (TAMs). Therefore, there would be significant false positive fluorescence in LNs due to FRβ from TAMs.

EpCAM as a biomarker for LN metastases may present an opportunity for use in IMI for precision LN dissection. Such a development would allow for the cancer surgeon to dissect all positive LNs and avoid leaving a false negative LN behind. Because there is minimal background fluorescence with EpCAM in negative LNs, EpCAM is a highly specific and sensitive target. Although EpCAM is a known epithelial tumor marker, this is the first study looking at EpCAM as a target for IMI of LNs with cancer cells.

Although the results with EpCAM are promising, limitations are acknowledged. Given the retrospective nature of the study and the utilization of formalin-fixed paraffin-embedded tissues, in vivo imaging could not be performed with the tissue available. Despite this, we used a reliable mAb and we achieved consistent results across a large sample size. Additionally, we believe the results to be reproducible given the standardized methodology with antibody-based immunofluorescence.

This preliminary study showed that EpCAM may be a promising marker of LNs with metastases, but due to ethical limitations with obtaining human LN specimens, only formalin-fixed paraffin-embedded tissues specimens were used in this study. Further investigation is needed using in situ, entire lymph nodes to determine the fluorescence and depth of penetration on a larger specimen.

In summary, EpCAM is a highly promising target for IMI in guided LN dissection in lung cancer surgeries. There was significant fluorescence in both hilar and mediastinal LNs, and in different primary tumor histologies. EpCAM showed strong specificity and low background fluorescence in all LN specimens, even at low tumor burdens. EpCAM may be useful for the surgical oncologist for intraoperative detection of positive LNs from epithelial cancers, including lung cancer, and this warrants further investigation with in vivo studies.

## Supplementary Information

Below is the link to the electronic supplementary material.

ESM1 (DOCX 14.6 KB)

## Data Availability

The authors confirm that the data supporting the findings of this study are available within the article and its supplementary materials.
