# Supplementary material for: Epithelial Cell Adhesion Molecule Is an Accurate Target for Fluorescence Guided Imaging of Lymph Nodes
Source: Mol Imaging Biol. 2025 Oct 14;27(6):984–92. doi: 10.1007/s11307-025-02058-5 (PMC12804259; doi:10.1007/s11307-025-02058-5)
Supplement: Supplementary file 1 — (DOCX 14.6 KB) [file 11307_2025_2058_MOESM1_ESM.docx]

**Supplemental Material**

Supplemental Table 1: Patient Characteristics

| Variables | N (%) |
| --- | --- |
| Age | 57.9 ± 11.4 |
| Sex  Male  Female | 55 (67.9)  26 (32.1) |
| Location  Hilar  Mediastinal | 23 (47.9)  25 (52.1) |
| Histology  Adenocarcinoma  Neuroendocrine | 42 (87.5)  6 (12.5) |
| Pathologic N Stage  N0  N1  N2  N3 | 0 (0)  23 (47.9)  25 (52.1)  0 (0) |
